# Elevated CO_2_ delays the early development of scleractinian coral *Acropora gemmifera*

**DOI:** 10.1038/s41598-018-21267-3

**Published:** 2018-02-12

**Authors:** Xiangcheng Yuan, Tao Yuan, Hui Huang, Lei Jiang, Weihua Zhou, Sheng Liu

**Affiliations:** 1 CAS Key Laboratory of Tropical Marine Bio-resources and Ecology, South China Sea Institute of Oceanology, Chinese Academy of Sciences, Guangzhou, 510000 China; 2 Guangdong Provincial Key Laboratory of Applied Marine Biology, South China Sea Institute of Oceanology, Chinese Academy of Sciences, Guangzhou, 510000 China; 3 Tropical Marine Biological Research Station in Hainan, Chinese Academy of Sciences, Sanya, 572000 China

## Abstract

The effects of elevated CO_2_ on the early life stages of coral were investigated by culturing the pelagic larvae and new recruits of *Acropora gemmifera* at three concentrations of CO_2_ (corresponding to pH = 8.1, 7.8 and 7.5, respectively). Acidified seawater resulted in fewer *A*. *gemmifera* larvae settling, and led to the production of smaller new recruits by slowing the development of the skeleton. The delayed development of new recruits due to elevated CO_2_ was consistent with the downregulation of calcification related genes. Several genes related to HCO_3_^−^ and Ca^2+^ transporters were downregulated by elevated CO_2_, with solute carriers (SLC) (membrane transport proteins) possibly playing an important role. The downregulation of these membrane transport proteins might suppress the transport of calcium, bicarbonate and organic matter, resulting in the delayed development of *A*. *gemmifera*.

## Introduction

Coral calcification is believed to be largely controlled by the degree of aragonite saturation (Ω_A_), and is significantly decreased by elevated atmospheric partial pressure CO_2_ (*p*CO_2_)^1–3^ The early life stages of many marine organisms seem to be particularly vulnerable to acidified seawater. Consequently, there is an increasing concern about the potential impacts of increasing *p*CO_2_ on corals during their early life stages^4^.

**Figure 1:**
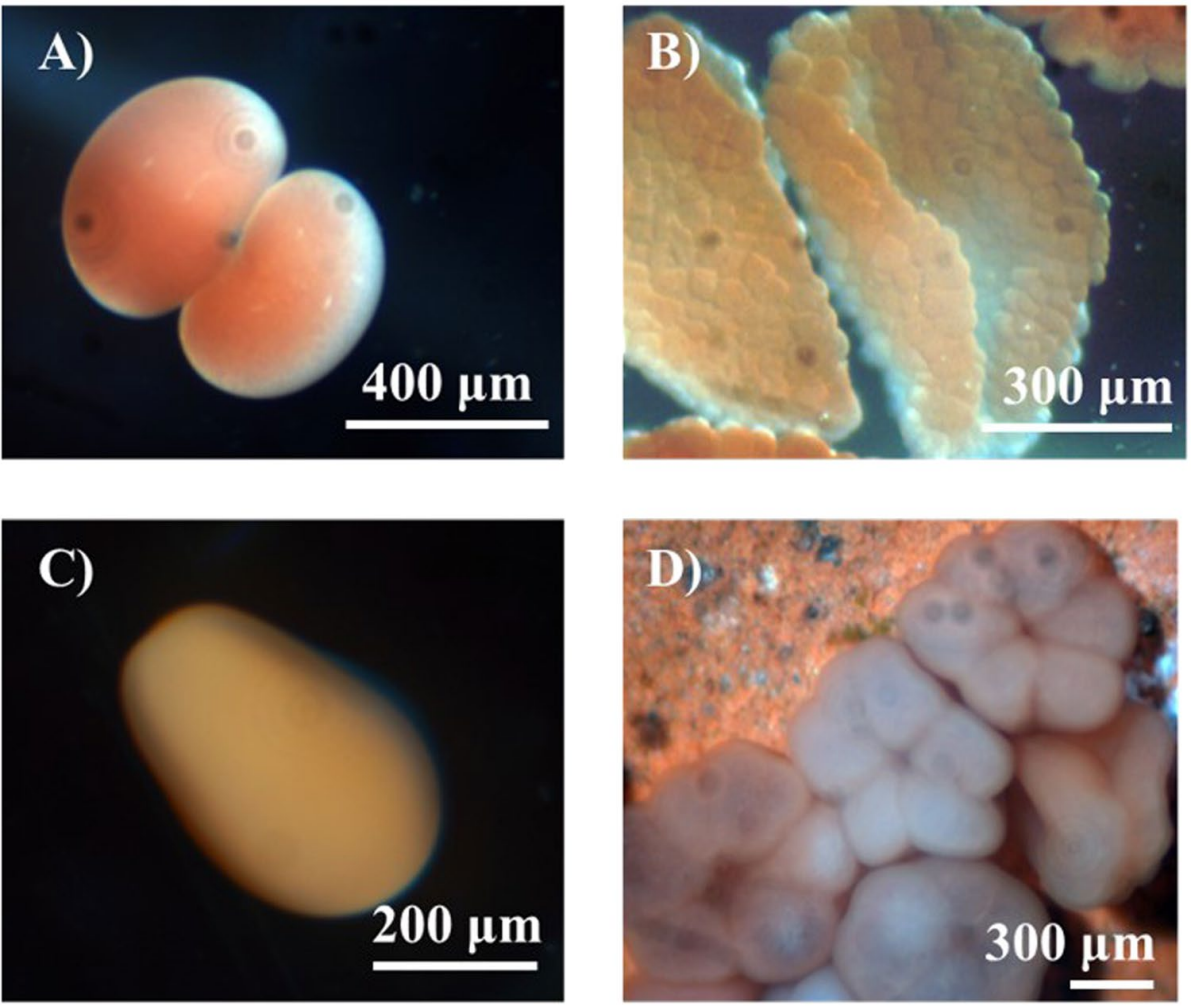
Embryonic development and larval development of coral *A*. *gemmifera* (From zoom stereo microscope). (**A**) 2 cleavage (2 hr), (**B**) larvae (prawn chip, 6 hr), (**C**) rodlike planula (47 hr), (**D**) settled new recruits (5 d).

There are both pelagic and benthic phases in the coral life-cycle^5^. The planktonic larvae of stony corals are non-calcifying until they settle, with a previous study documenting their morphological changes^6^ (Fig. 1). After coral larvae settle, new recruits develop a calcifying base, and form a synapticular ring and septa to establish the structure of the coral skeleton. The skeletal morphology changes rapidly during the early life stage, suggesting that the expression of calcification related genes also changes^7^. Coral growth and calcification are closely related to various protein transporters of Ca^2+^, H^+^ and HCO_3_^− ^^8–11^, as well as coral skeleton organic matrix proteins (SOMPs)^7,12,13^. There has been detailed information on the settlement and metamorphosis of early life stages of coral^14,15^. In addition, previous studies reported that high CO_2_ affected the early life stage of coral, by delaying calcification and settlement^16–20^, modulating metabolism^21,22^, changing skeleton morphology^19,23^, and reducing survival and algal infection rates^24^. Also, some studies showed that elevated CO_2_ exhibited no significant effects on coral survival and corallite area^14,25^.

Gene expression has been analysed to understand the post-settlement success and skeleton formation of coral during the entire period of early development^26,27^. Moya, *et al*.^28^ used the Illumina RNAseq approach to study how acute exposure to elevated CO_2_ affected the gene expression of the early life of *Acropora millepora*. The authors reported that the expression of most ion transport proteins was not affected by elevated CO_2_, while many membrane-associated or carbonic anhydrases were downregulated. Although corals lacked a strong response to elevated *p*CO_2_ in the study of Rocker, *et al*.^29^, new recruits might have the capacity to acclimate rapidly to elevated pCO_2_ by upregulating specific heat shock proteins (HSPs) and a suite of anti-apoptotic proteins (e.g. Bcl-2 family members)^30^. Kaniewska, *et al*.^31^ also demonstrated the upregulation of membrane transporters under high CO_2_ concentrations, as well as the regulation of genes involved in the cytoskeletal interactions and cytoskeletal remodelling of membranes. The expression of heat shock proteins, carbonic anhydrase, and rubisco protein has also been studied in coral larvae^32–34^, supporting the assumption that elevated temperature and CO_2_ are impacting coral development.

Previous studies primarily used *A. millepora* as a model species in the analysis of gene expression. However, studies of other species might provide a more complete understanding of how coral communities respond to high CO_2_ concentrations. The rapidly growing branching coral, *A. gemmifera*, is widely distributed, and is an ecologically important genus in the South China Sea. In the current study, the pelagic larvae and new recruits of *A. gemmifera* were exposed to three *p*CO_2_ concentrations to investigate the transcriptome of the coral during the early life stages, with a focus on the adhesion and structural proteins involved in calcification. In previous studies, sequencing reads were usually mapped onto a transcriptome assembly of an *A. millepora*. The present study conducts a transcriptome analysis using *de novo* RNA-seq and gene expression analysis to gain deep insight into the responses of calcification related genes to climate changes.

## Results

### Settlement, survivorship, and morphological change

**Figure 2:**
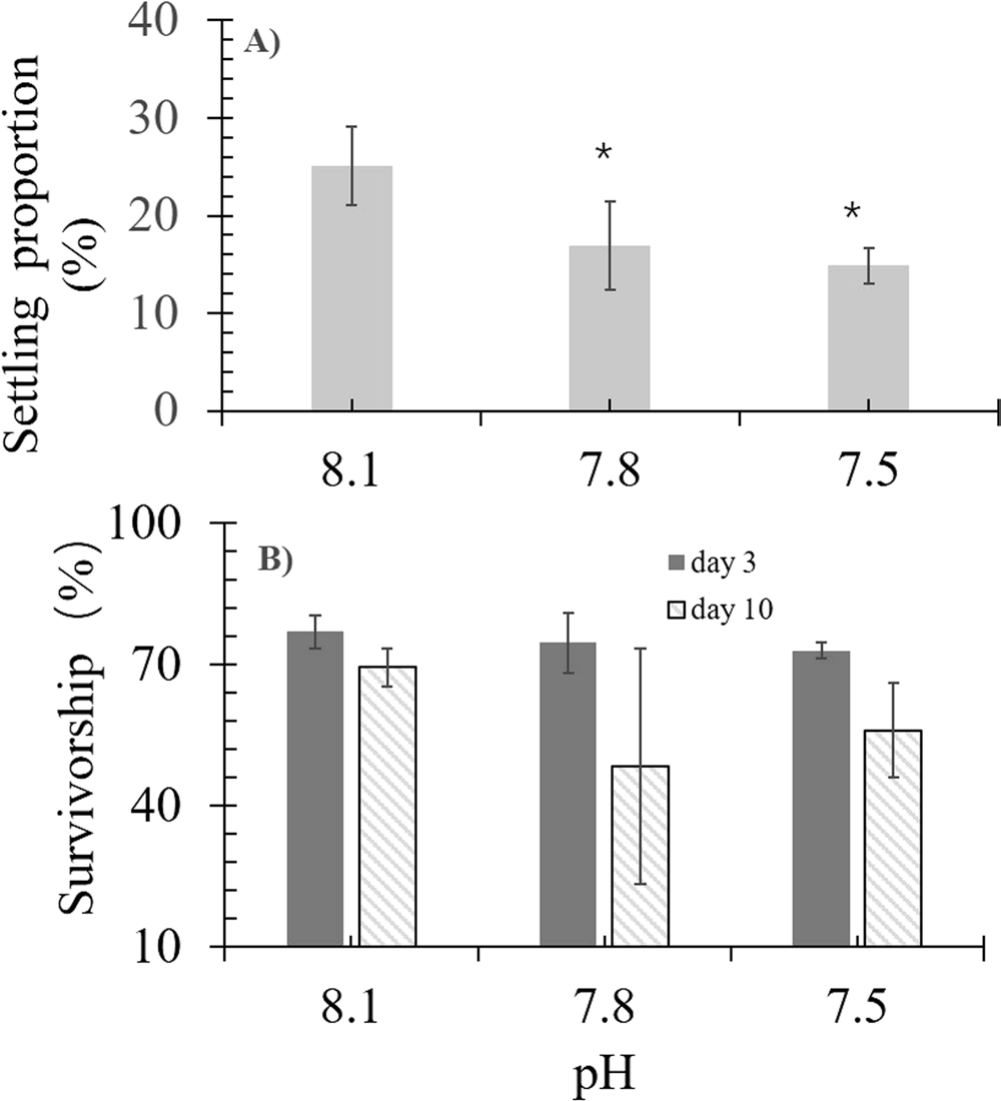
(**A**) The larval settlement ratios; and (**B**) survivorship of new recruits after 3-day and 10-day settlement at different pH values. *Denotes significant difference (p < 0.05), compared to the control (pH = 8.1).

**Figure 3:**
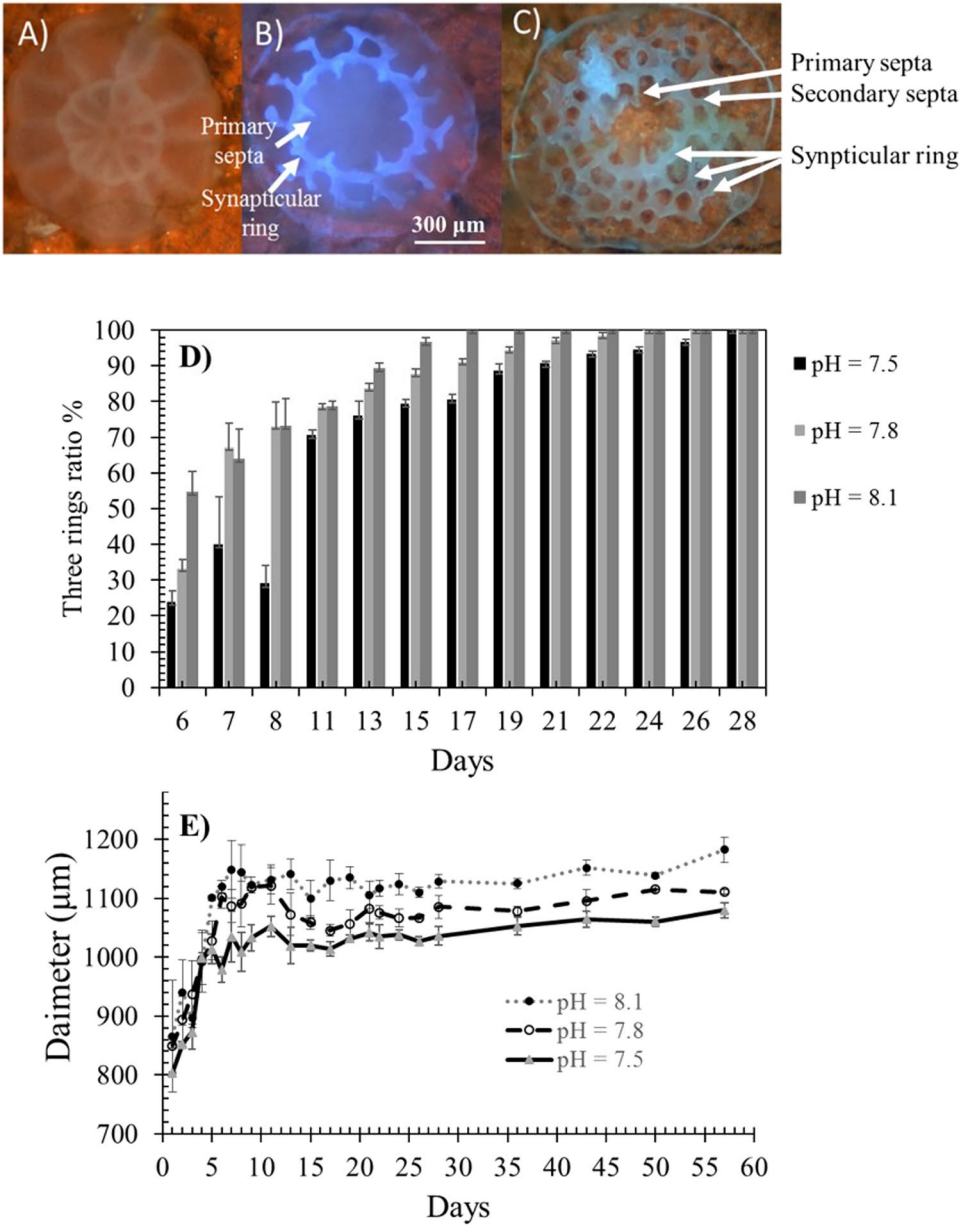
Progressive morphological changes in *A. gemmifera*: (**A**) first day settlement; (**B**) formation of first synapticular ring and primary septa on the 2–7th days; (**C**) three synapticular rings with secondary septa and theca after 5 days; (**D**) the ratios of new recruits showing three synapticular rings; and (**E**) average diameter measurement of *A. gemmifera* (n = 3) at pH = 8.1, 7.8 and 7.5 respectively).

The settling proportion was lower in low pH treatments (p < 0.05), whereas survivorship exhibited no significant difference between the treatments (p > 0.05) (Fig. 2). Tank effects were not significant (p > 0.05). The first synapticular ring and primary septa in new coral recruits were detected after approximately 3 days after settlement. Three synapticular rings with secondary septa and theca were observed after 5 days (Fig. 3A–C). For treatments in which pH was 7.8 and 7.5, the ratios of new recruits with three synapticular rings were always lower than those in the control (Fig. 3D). All settled new recruits had more than three synapticular rings after 17 days at pH 8.1, with skeletal development being visibly suppressed at lower pH (Fig. 3D).

**Figure 4:**
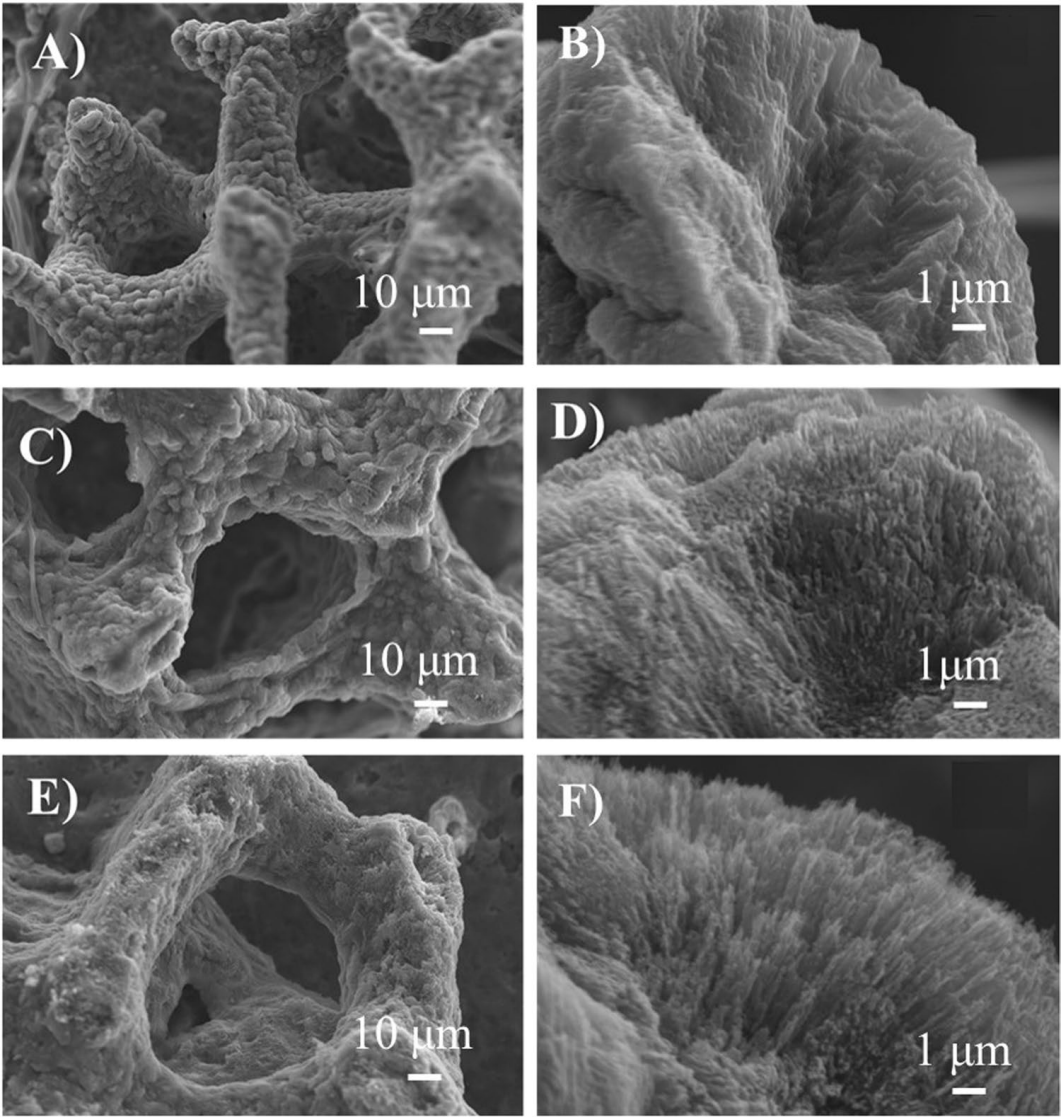
The skeletal structure on the 40th day after settlement (**A**) and (**B**) at pH = 8.1; (**C**) and (**D**) at pH = 7.8; and (**E**) and (**F**) pH = 7.5 by scanning electron microscopy imaging. In (**A**), (**C**) and (**E**) scale bar represents 10 µm, in (**B**), (**D**) and (**F**) scale represents 1 µm.

From 5 days after settlement, the diameter of the new recruits remained significantly smaller in the treatment with lower pH than the control (p < 0.05) (Fig. 3E). In addition, crystals were granule-like at pH 8.1, whereas crystals became increasingly distorted and needle-like in treatments with lower pH (Fig. 4A–F).

### Differentially expressed genes

**Figure 5:**
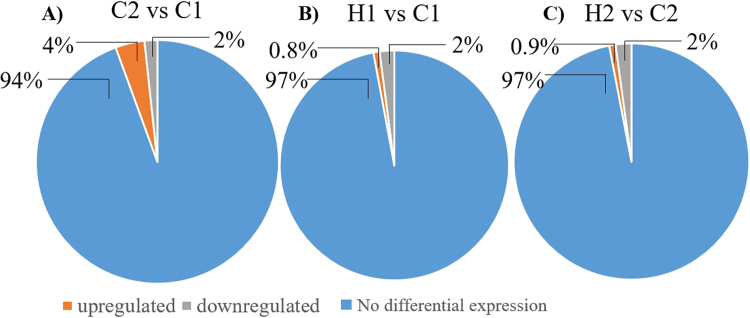
The percentage of differentially expressed genes. (**A**) Control after 40 days vs 3 days (C2 vs C1), (**B**) high CO_2_ treatment after 3 days vs control after 3 days (H1 vs C1), (**C**) high CO_2_ treatment after 40 days vs control after 40 days (H2 vs C2).

A total of 12,367,906 reads were obtained from the *A. gemmifera* new coral recruits, with ~60% of reads being mapped to the total *de novo* unigene assembly (Table S1). As new recruits developed, only ~6% of transcripts were differentially expressed between C1 (3 days after settlement) and C2 (40 days after settlement) (Fig. 5). In addition, ~3% transcripts were differentially expressed in both acute (3 days) and prolonged treatments (40 days) with elevated CO_2_ (Fig. 5).

Significant mortality of new recruits was observed in the treatments with elevated CO_2_ (Fig. 2B). Some apoptosis related genes were downregulated (Table 1) in response to elevated CO_2_, including apoptosis regulator BCL-2, caspase-3 and caspase-8. Some of these apoptosis-related genes are responsible for the negative regulation of apoptotic processes, while others might positively regulate apoptotic processes (Table 1).

**Table 1:** Differential expressions of apoptosis-related genes between the control in 40 days vs. control in 3 days after settlement (C2 vs. C1), the acidified treatment in 3 days vs. control in 3 days after settlement (H1 vs. C1), and acidified treatment in 40 days vs. control in 40 days after settlement (H2 vs. C2). All data were filtered with p < 0.0001 and FDR <0.0001.

Accession	Definition	Nr-Evalue	C2 vs C1	H1 vs C1	H2 vs C2
XM_015899700.1	apoptosis regulator BCL-2; negative regulation of apoptotic process	0	−2.18	−2.64	−5.83
XM_015900639.1	BCL2-associated athanogene 3; negative regulation of apoptotic process	2.00E-127	1.57	—	−2.51
XM_015919947.1	caspase-3-like; apoptotic signaling pathway	1.00E-72	—	−1.32	—
XM_015905634.1	caspase 8; positive regulation of apoptotic process	0	−1.38	−3.42	−2.51

A series of genes related to the HCO_3_^−^ and Ca^2+^ transporters were downregulated in response to elevated CO_2_ (Table 2). Solute carriers (SLC) are membrane transport proteins located in the cell membrane that transport charged and uncharged inorganic and organic molecules. Solute carrier (SLC) genes were mostly upregulated after 40 days compared to 3 days (C2 vs C1), and were mostly downregulated in response to elevated CO_2_ after 40 days of settlement compared to 3 days of settlement (H1 vs C1 or H2 vs C2). SLC4 member 10 is responsible for transporting HCO_3_^−^ (Table 2), while SLC8 and SCL24 are Ca^2+^ transporters. The plasma membrane Ca^2+^ transporting ATPase was also downregulated in response to elevated CO_2_ (Table 2). Many other members of various SLC families were also downregulated by the high CO_2_ treatment. For example, SLC12 and SLC25 were downregulated after 40 days under high CO_2_ (Table 2). Similarly, some skeleton organic matrix genes were downregulated by the high CO_2_ treatments, including cadherin, thrombospondin, hemicentin, actin, collagen, and galaxin after 3 days of settlement (Table 3).

**Table 2:** Differential expressions of ion transporter-related genes between the control in 40 days vs. control in 3 days after settlement (C2 vs. C1), the acidified treatment in 3 days vs. control in 3 days after settlement (H1 vs. C1), and acidified treatment in 40 days vs. control in 40 days after settlement (H2 vs. C2). All data were filtered with p < 0.0001 and FDR <0.0001.

KEGG entry	Definition	KEGG-Evalue	C2 vs C1	H1 vs C1	H2 vs C2
tad:TRIADDRAFT_54168	SLC4 (anion exchanger), member 2	1.00E-39	1.99	—	—
spu:587119	SLC4 (anion exchanger), member 3;	0	3.33	—	—
cfa:478766	SLC4 (sodium bicarbonate transporter), member 10	0	—	−1.93	−1.61
nve:NEMVE_v1g127372	SLC9 (sodium/hydrogen exchanger), member 3	2.00E-101	1.17	−1.78	—
nve:NEMVE_v1g166011	Ca^2+^ transporting ATPase, plasma membrane	0	1.79	−1.00	−1.04
nve:NEMVE_v1g239709	SLC8 (sodium/calcium exchanger)	2.00E-113	—	−1.61	−1.44
mdo:100023316	SLC24 (sodium/potassium/calcium exchanger), member 1	5.00E-81	−3.87	—	—
nve:NEMVE_v1g206699	SLC24 (sodium/potassium/calcium exchanger), member 2	8.00E-52	—	−3.42	−3.51
dre:100334346	SLC24 (sodium/potassium/calcium exchanger), member 4	2.00E-111	—	−1.34	—
nve:NEMVE_v1g80232	SLC26 (sodium-independent sulfate anion transporter), member 11	2.00E-115	2.12	—	—

**Table 3:** Differential expressions of skeletal organic matrix-related genes between the control in 40 days vs.

KEGG entry	KEGG Orthology	Definition	C2 vs C1	H1 vs C1	H2 vs C2
dre:407978	K06813	cadherin 23	1.11	1.30	—
ptr:457086	K04601	cadherin EGF LAG seven-pass G-type receptor 2 (flamingo)	−2.08	−1.95	—
tad:TRIADDRAFT_62266	K04600	cadherin EGF LAG seven-pass G-type receptor 1 (flamingo)	—	−1.51	—
dre:368436	K04600	cadherin EGF LAG seven-pass G-type receptor 1 (flamingo)	—	−1.42	−1.63
cin:100169670	K04659	thrombospondin	—	—	−1.63
bfo:BRAFLDRAFT_56687	K05692	actin beta/gamma 1	—	−1.07	—
cfa:475371	K13956	actin-binding protein IPP	−1.64	—	—
acs:100556853	K06236	collagen, type I/II/III/V/XI, alpha	−9.25	−1.06	−1.05
aml:100478752	K06237	collagen, type IV, alpha	—	−3.90	−1.05
acs:100558915	K06238	collagen, type VI, alpha	−1.38	—	1.95
xtr:100495209	K08132	collagen, type XII, alpha	—	−1.29	—
acs:100562332	K06823	collagen, type XVIII, alpha	−1.84	—	—

control in 3 days after settlement (C2 vs. C1), the acidified treatment in 3 days vs. control in 3 days after settlement (H1 vs. C1), and acidified treatment in 40 days vs. control in 40 days after settlement (H2 vs. C2). All data were filtered with p < 0.0001 and FDR < 0.0001.

## Discussion

### Survivorship and regulators of apoptosis

The survivorship of new recruits decreased after 40 days of exposure to high CO_2_ concentrations, but not significantly (Fig. 2B). As previous studies showed that the growth rates and colony size of hermatypic corals are inversely proportional to mortality risk^35,36^, the slow development of skeleton synapticular rings under elevated CO_2_ may potentially increase the risk of morality. In addition, suppressed growth under elevated CO_2_ is unfavourable for new recruits that compete for space with algae and other benthic organisms, because it reduces the fitness of new recruits, affecting population dynamics^37^.

Previous studies showed that the expression of apoptotic genes is generally related to cellular mortality and survivorship, with the expression being either pro- or anti-apoptosis^30^. Caspase 8 is essential for programmed cell death, and was downregulated in the high CO_2_ treatments of the current study. In addition, anti-apoptotic proteins (e.g. Bcl-2 family members) were also downregulated (Table 1), whereas heat shock proteins (HSPs) and heat shock factors (HSFs) were not differentially expressed (data not shown). A previous study showed that new recruits of *A. millepora* could acclimate to elevated *p*CO_2_ by upregulating the expression of HSPs, as well as Bcl-2 family members^30^; however, the current study indicated that this mechanism does not exist in *A. gemmifera*. Thus, different coral species might trigger different molecular mechanisms in responses to elevated *p*CO_2_.

### Delayed development and calcification related genes

In this study, the settlement, diameter, skeleton structure, and crystal shape of new recruits were affected by elevated CO_2_ concentrations. These results were consistent with previous studies where new recruits reared in OA had smaller diameters than did the control^38–40^. The current study showed that, in addition to diameter, the ratio of new recruits with three synapticular rings was lower before 28 days under higher CO_2_ concentrations. To our knowledge, only a few studies demonstrate the effects of elevated CO_2_ on the development of skeleton synapticular rings^23^. The slow development of the coral skeleton prompted us to conduct a more extensive survey of the major genes related to calcification, and to investigate their expression levels. Two major components contribute to the calcification process: (1) transporters of Ca^2+^, H^+^ and inorganic carbon, and (2) organic matrix proteins that catalyse the crystals to form macroscopic structures^41^.

Various transporters of Ca^2+^, H^+^ and inorganic carbon have been examined by previous studies^9–11^. Among these transporters, Ca^2+^ transporting ATPase was presumably a transporter of Ca^2+^ and H^+ ^^9^. In our study, Ca^2+^ transporting ATPase was only differentially expressed during the first 3 days under high CO_2_ concentrations, but was downregulated after 40 days of exposure. Carbonic anhydrases contribute to the interconversion of carbon dioxide and bicarbonate^11^, which were also not differentially expressed under high CO_2_ in this study.

In addition, solute carriers (SLC) are membrane transport proteins located in the cell membrane that transport charged and uncharged inorganic and organic molecules. Zoccola, *et al*.^42^ reported that the SLC4 and SLC26 families are both bicarbonate anion transporters in the coral *Stylophora pistillata*. SLC4 (anion exchanger) member 2 was neither differentially expressed in response to elevated CO_2_ in *A. millepora*^28^ nor *A. gemmifera* (this study); however, SLC4 member 10 (bicarbonate anion transporter) was downregulated in under high CO_2_ concentrations in the current study (Table 2). This result suggests that the bicarbonate transports were suppressed by elevated CO_2_, with SLC4 member 10 either supplying bicarbonate anion at the site of calcification or aiding in pH regulation^42^. A previous study showed that SLC26 was differentially expressed by different temperatures^43^; however, SLC26 member 11 was not differentially expressed by high CO_2_ in our study (Table 2). Hence, elevated CO_2_ might reduce bicarbonate anion transport by suppressing the expression of SLC4 member 10 rather than SLC26. In addition to bicarbonate anion transport, the calcium transporters (SLC8 and SLC24) were also downregulated by elevated CO_2_, including SLC 24 members 2 and 4 (Table 2). The members of the SLC24 gene family encode K^+^-dependent Na^+^/Ca^2+^ exchangers (NCKX) that utilise both inward Na^+^ and outward K^+^ gradients to extrude Ca^2+^ from cells^44^. However, it remains unclear whether SLC24 is located on the membrane of tissue cells or the calicoblastic cells. Consequently, it is also unclear whether SLC24 is able to regulate coral calcification. Both HCO_3_^−^ and Ca^2+^ transporters were downregulated under elevated CO_2_ concentrations, which probably led to slower calcification rates and the delayed development of new recruits. In addition to HCO_3_^−^ and Ca^2+^, the transporters of organic matter (such as oligopeptides, monocarboxylic acid, fatty acids, and amino acids) were also downregulated (Table S2). The presence of fewer organic matter transporters might decrease the energy supply available for the calcification and growth of coral.

It has been long hypothesized that the precipitation of aragonite is catalysed by and organized on an extracellular organic matrix. A recent study identified 36 coral of coral skeleton organic matrix proteins (SOMPs) in *S. pistillata*^12^. In our study, only five out of these 36 skeletal proteins were differentially expressed, including cadherin, thrombospondin, hemicentin, actin, and collagen (Table 3). However, these proteins were usually downregulated in H1 vs. C1, but not in H2 vs C2. The specific functions of some organic matrix proteins have yet to be determined in coral species, but their functions have been studied in human and other animals. Cadherin plays an important role in cell adhesion, forming junctions to bind cells together within tissues^45^. Collagens are also important in the regulation of cell-cell adhesion, differentiation, and wound healing^46^. Coral acid-rich protein (CARPs) members have been reported as putative calcification-related proteins in *A. millepora*^13^ and *S. pistillata*^12,47^, but were not differentially expressed in response to elevated CO_2_ in our study (data not shown).

## Conclusions

The present study explored the effects of elevated *p*CO_2_ on the post-settlement development of new coral recruits of *A. gemmifera*. Elevated CO_2_ slowed the formation of the skeleton structure and crystal microstructure of new recruits, slowing settlement and reducing the diameter of new recruits. Many solute carriers (SLC) (membrane transport proteins) were downregulated in response to elevated CO_2_, as well as the SLC4 family members responsible for supplying HCO_3_^−^ to the site of calcification. The downregulation of membrane transporters proteins might represent the mechanism underlying the delayed development of new coral recruits.

## Materials and Methods

### Sampling and incubation

In April 2015, 5 colonies of *A. gemmifera* (~40 cm diameter) were collected from Luhuitou fringing reef, Sanya Bay, China (18° 15′ N; 109° 25′ E) at ~2–5 m depth. Before spawning, colonies were transferred to outdoor flow-through aquaria in Sanya Bay, Hainan Island, outside the Tropical Marine Biological Research Laboratory. Ten colonies of *A. gemmifera* (~40 cm diameter) were collected. These colonies were >5 m away from each other, assuming their genotypes were not the same. Gametes were collected from these 10 colonies without separate crossings.

In our CO_2_ system, seawater chemistry was manipulated by continuous and direct bubbling of pure CO_2_ (except for the control treatment). The bubbling rates were controlled by high precision pressure gauges and valves (DC01-01, Dici, China) and pH controller (pH2010, WEIPRO, China) to create the desired pCO_2_ (Table 4). Seawater was pumped from the reef water (~200 m away from our culture tank) at a depth of 5 m and run through a sand- filter. As the seawater was not further filtered, no extra food and zooxanthellae were added into culture aquaria to create. Each aquarium was covered with a screen to provide a light field that was approximately 50% of the surface solar irradiance, a condition similar to that at a depth of 3–5 m. Treated seawater was then pumped to the 90 L experimental tanks (three replicate tanks per treatment) via PVC-pipes. The water-exchange rate was 600 ± 10 mL min^−1^ (Fig. S1). The tanks were partially immersed in a 2000 L flow-through aquarium.

**Table 4:** Values of pH, temperature (T), salinity, total alkalinity (TA), dissolved inorganic carbon (DIC), *p*CO_2_ and aragonite saturation state (Ω_A_) during incubations. All values are means ± SD.

pH	T (°C)	Salinity	TA (μmol L^−1^)	DIC (μmol L^−1^)	*p*CO_2_ (μatm)	Ω_A_
8.1 ± 0.14	27 ± 2	34 ± 0.02	2219 ± 26	1902 ± 32	389 ± 24	3.4 ± 0.06
7.8 ± 0.16	27 ± 2	34 ± 0.02	2225 ± 45	2021 ± 87	700 ± 75	2.4 ± 0.08
7.5 ± 0.18	27 ± 2	34 ± 0.02	2224 ± 31	2110 ± 96	1214 ± 102	1.6 ± 0.10

Larvae were cultivated in flow-through tanks (90 L) at a density of ~1 larvae/ml under ambient conditions (pH NBS 8.16 ± 0.01, 27.5 °C, total alkalinity (TA) 2250.8 ± 5 μM, salinity 33 psu), ~750 ppm CO_2_ (pH = 7.8) or 1200 ppm CO_2_ (pH = 7.5) (Table 4), reflecting the control condition, intermediate and high CO_2_ concentrations based on IPCC predictions for the 21st century (2007). There were four replicates in each treatment, and the turnover time of seawater of each tank was 12 hours. The outflow of each tank was covered with a 180 μm mesh to avoid larvae loss. About 10–15 preconditioned terrocota tiles with a mixed community of crustose coralline algae were placed into each tank to induce larval settlement. After four days, larvae were randomly sampled for settlement assays. Six days later, all swimming larvae were flushed away, and new recruits on tiles were reared in continuous flow-through tanks for observing survivorship, diameter, and skeleton structure (90 L) (Fig. S1). The survivorship of new recruits on tiles was assessed on the 3rd and 10th days after settlement, by observing it using a stereomicroscope (Olympus, SZ51). Death was defined as the time point when live tissue was no longer present. Survivorship was expressed as the proportion surviving within each tank. The number of new recruits was counted, and image of new recruits was taken using a stereomicroscope. The diameter of live tissue was measured with the program ToupView 3.7. The settled recruits were collected at the end of 40-day incubation, and their skeletons was examined by scanning electron microscopy (SEM, ZEISS Supra55).

### Settling proportion

The settling proportion was estimated in a separate experiment. In this separate experiment, about 300 larvae were sampled from each treatment tank and transferred to lidded chambers (10 L) filled with seawater with the corresponding experimental treatments. A preconditioned terrocota tile with a mixed community of crustose coralline algae was placed at the bottom of the chambers. Half of the seawater in each chamber was replaced every 12 hours. Larval settlement was assessed after two days using a stereomicroscope (Olympus, SZ51). Settling proportion was estimated by the number of new recruits/total initial larvae.

### TA and pH

Water samples were collected every three days from each tank, and total alkalinity (TA) was measured by an open-cell automatic titrator (Metrohm 877 titrino plus) using 0.1 M HCl for each 70 g seawater sample. Accuracy of the measurements was checked against certified seawater reference material (Batch 118) obtained from Dr A. Dickson. The pH in culture tanks was continuously monitored using a Ross semi-micro glass combination pH electrode (Orion). The other carbonate system parameters (i.e. dissolved inorganic carbon (DIC), Ω and pCO_2_) were calculated using a spreadsheet version of the CO2SYS program^48^, based on measurements of temperature, salinity, pH and alkalinity. The calculation used the CO_2_ equilibrium constants K1 and K2^49^.

### *De novo* transcriptome sequencing

On the 3rd and 40th day after settlement, settled new recruits (~10–50 new recruits per biological sample) of each tank were randomly sampled from ~10–15 tiles with a sterile scalpel, and 1 sample per experimental treatment tank were obtained. The samples of control and high CO_2_ treatments (1200 μatm CO_2_) were snap frozen the samples in liquid nitrogen and then stored the samples at −80 °C until further processing. TRI-Reagent (Sigma) was used for RNA extraction according to the manufacturer’s manual. Samples were stored at −80 °C, until transcriptome sequencing was carried out for *de novo* assembly and analysis by the Huada Gene Company (http://www.genomics.cn/index).

After the mRNAs were fragmented into short fragments as templates, double-stranded cDNA was synthesized. The ligated fragments were then generated by a series of reaction processes that included purification of PCR products, end repair, and ligation of Illumina adapters. AMPure XP system was used for the purification PCR products, and the Agilent Bioanalyzer 2100 system was used to assess the library quality. An Illumina HiSeq™ 2000 sequencing platform was employed to sequence the cDNA library (NEB, USA).

### Data filtering, *de novo* assembly and annotation

We filtered the raw data to generate clean reads by removing adapter sequences, reads with unknown bases (N) >10%, and low-quality sequences (the percentage of low quality bases of quality value <5). After obtaining clean reads, the clean reads were *de novo* assembled by the Trinity program into contigs^50^. The read coverage in each sample roughly exhibited a similar distribution. All unigenes were annotated using the BLASTx against the NCBI Nr (http://www.ncbi.nlm.nih.gov/genbank/), and KEGG pathway (http://www.genome.jp/kegg/pathway.html) databases, with an E-value cut-off of 1E-5. To further analyze the annotation results, the GO function of unigenes were classified in terms of molecular function, biological process, as well as cellular component (http://www.geneontology.org/) using Blast2GO^51^.

### Differential expression analysis

Clean reads of each treatment were mapped to reference genes transcriptome generated by total *de novo* RNA-seq^52,53^. The gene expression level was calculated by the value of FPKM (Fragments Per Kilobase of transcript per Million). The differentially expressed genes were annotated using GO and KEGG enrichment analyses according to a method similar to that described by^52,53^. The best BLAST hit was used to assess differential gene expression, which was estimated with log_2_fold change. The triplicate samples were compared between control 3 days after settlement (C1), control 40 days after settlement (C2), acidified treatment 3 days after settlement (H1), and acidified treatment 40 days after settlement (H2).

### Statistical analyses

Two-way analyses of variance (ANOVA) tested the effects of *p*CO_2_ and different tanks on coral biological parameters (e.g. settling proportion, survivorship, diameter, and gene expression). Different treatments of *p*CO_2_ and different tanks were fixed, whereas each coral biological parameter was a random factor. Post hoc Tukey tests were conducted if significant effects were obtained (p < 0.05). Statistical analyses were performed using SPSS 19 System for Windows. The differential gene expression analysis of the two experimental groups (high CO_2_ treatment vs control) was determined using Expdiff. A q value (or FDR) of <0.005 & |log_2_fold change|>1 was set as the threshold for significantly different expressions.

### Availability of raw data

The raw data used for assembly, annotated expression data and annotated data were deposited in the National Center for Biotechnology Information (NCBI) Gene Expression Omnibus (GEO) under accession number GSE96935.

## Electronic supplementary material

Supplementary Information
