# Supplementary material for: Elevated CO2 delays the early development of scleractinian coral Acropora gemmifera
Source: Sci Rep. 2018 Feb 12;8:2787. doi: 10.1038/s41598-018-21267-3 (PMC5809585; doi:10.1038/s41598-018-21267-3)
Supplement: Supplementary file 1 — Supplementary Information [file 41598_2018_21267_MOESM1_ESM.pdf]

Elevated CO_2 delays the early development of scleractinian coral *Acropora gemmifera*

Xiangcheng Yuan, Tao Yuan, Hui Huang, Lei Jiang, Weihua Zhou, Sheng Liu

Tab. S1. Summary statistics of the transcriptome assembly for the triplicate samples in control after 3 days (C1-1 to C1-3), control after 40 days (C2-1 to C2-3), acidified treatment after 3 days (H1-1 to H1-3), and acidified treatment after 40 days (H2-1 to H2-3)

Treatment	Sample ID	Total Reads	Total Base Pairs	Total Mapped Reads
control 3 days	C1-1	12,367,906	606,027,394	7,919,003(64.03%)
	C1-2	11,904,632	583,326,968	7,621,190(64.02%)
	C1-3	11,782,573	577,346,077	6,764,935(57.41%)
control 40 days	C2-1	12,241,461	599,831,589	5,234,034(42.76%)
	C2-2	12,254,767	600,483,583	6,564,457(53.57%)
	C2-3	12,270,456	601,252,344	7,150,203(58.27%)
high CO_2 3 days	H1-1	11,789,649	577,692,801	7,028,455(59.62%)
	H1-2	11,722,947	574,424,403	7,560,269(64.49%)
	H1-3	12,493,366	612,174,934	8,181,842(65.49%)
high CO_2 40 days	H2-1	11,970,508	586,554,892	7,405,616(61.87%)
	H2-2	11,658,887	571,285,463	7,163,855(61.45%)
	H2-3	12,388,176	607,020,624	7,397,138(59.71%)

Tab. S2 Annotated differentially expressed SLC genes other than calcium and bicarbonate transporters between the acidified treatment after 3 days vs. control after 3 days (H1 vs. C1), and acidified treatment after 40 days vs. control after 40 days (H2 vs. C1). Differential expression is estimated with log_2fold change. All data were filtered with $p < 0.0001$ and FDR< 0.0001.

KEGG entry	KEGG Orthology	Definition	C2 vs C1	H1 vs C1	H2 vs C2
acs:100562038	K05614	SLC1 (glial high affinity glutamate transporter), member 3;	1.01		
nve:NEMVE_v1g234329	K14347	SLC10 (sodium/bile acid cotransporter), member 7		-1.50	1.57
xla:100037166	K10951	SLC12 (sodium/potassium/chloride transporter), member 2;		-2.16	
nve:NEMVE_v1g104399	K10951	SLC12 (sodium/potassium/chloride transporter), member 2			-1.20
spu:586262	K14429	SLC12 (potassium/chloride transporters), member 9	1.07		
nve:NEMVE_v1g102562	K14429	SLC12 (potassium/chloride transporters), member 9		-2.83	-3.05
nve:NEMVE_v1g89298	K14428	SLC12 (potassium/chloride transporters), member 8		-1.79	
tad:TRIADDRAFT_30870	K14206	SLC15 (oligopeptide transporter), member 1		-3.53	
nve:NEMVE_v1g173993	K14638	SLC15 (peptide/histidine transporter), member 3/4	3.95	-1.64	1.65
xla:380349	K08180	SLC16 (monocarboxylic acid transporters), member 3	-1.34		-1.08
nve:NEMVE_v1g88648	K08187	SLC16 (monocarboxylic acid transporters), member 10;	2.77	2.97	-1.92
hmg:100213774	K08187	SLC16 (monocarboxylic acid transporters), member 10		1.34	
nve:NEMVE_v1g231086	K08193	SLC17 (sodium-dependent inorganic phosphate cotransporter)		-2.83	-7.13
nve:NEMVE_v1g124454	K14610	SLC19 (thiamine transporter), member 2/3		-1.83	
nve:NEMVE_v1g204860	K08145	SLC2 (facilitated glucose transporter), member 8			-1.10
nve:NEMVE_v1g228429	K08150	SLC2 (myo-inositol transporter), member 13		-1.35	
mgp:100543293	K08212	SLC22 (organic cation transporter), member 16		-1.19	
nve:NEMVE_v1g109102	K08202	SLC22 (organic cation transporter), member 4/5	2.08		

hmg:100209245	K08202	SLC22 (organic cation transporter), member 4/5;			-1.22
nve:NEMVE_v1g164090	K14611	SLC23 (nucleobase transporter), member 1/2	2.82		
nve:NEMVE_v1g164090	K14611	SLC23 (nucleobase transporter), member 1/2	3.23		
nve:NEMVE_v1g196629	K15116	SLC25, member 33/36	1.09		
nve:NEMVE_v1g184535	K15105	SLC25 (mitochondrial aspartate/glutamate transporter), member 12/13		-1.50	
nve:NEMVE_v1g242628	K15109	SLC25 (mitochondrial carnitine/acylcarnitine transporter), member 20/29			5.38
spu:588410	K14684	SLC25 (mitochondrial phosphate transporter), member 23/24/25/41		1.52	
hmg:100201695	K15107	SLC25 (mitochondrial glutamate transporter), member 18/22			-1.11
nve:NEMVE_v1g172833	K14684	SLC25 (mitochondrial phosphate transporter), member 23/24/25/41;			-1.42
nve:NEMVE_v1g236734	K15119	SLC25, member 39/40			2.76
hmg:100203878	K15121	SLC25, member 44;			-1.13
tad:TRIADDRAFT_60775	K14708	SLC26 (sodium-independent sulfate anion transporter), member 11	1.26	-1.26	
nve:NEMVE_v1g98096	K08745	SLC27 (fatty acid transporter), member 1/4	2.05		
tca:660540	K08745	SLC27 (fatty acid transporter), member 1/4		-1.06	
nve:NEMVE_v1g212057	K14210	SLC3 (neutral and basic amino acid transporter), member 1		-1.42	
nve:NEMVE_v1g214632	K15015	SLC32 (vesicular inhibitory amino acid transporter)	1.29	-1.47	
nve:NEMVE_v1g96724	K15015	SLC32 (vesicular inhibitory amino acid transporter)	1.56		
nve:NEMVE_v1g241391	K15015	SLC32 (vesicular inhibitory amino acid transporter)		-1.31	-1.73
nve:NEMVE_v1g96724	K15015	SLC32 (vesicular inhibitory amino acid transporter)		-1.21	
nve:NEMVE_v1g32113	K14683	SLC34 (sodium-dependent phosphate cotransporter)			-1.86
nve:NEMVE_v1g191532	K15272	SLC35 (UDP-sugar transporter), member A1/2/3	1.08		

nve:NEMVE_v1g214792	K15272	SLC35 (UDP-sugar transporter), member A1/2/3	1.13		
nve:NEMVE_v1g174894	K15287	SLC35, member F1/2	1.25		
acs:100552482	K13783	SLC37 (glycerol-3-phosphate transporter), member 1/2		-1.22	
rno:500973	K13783	SLC37 (glycerol-3-phosphate transporter), member 1/2		-1.11	
nve:NEMVE_v1g83628	K14996	SLC38 (sodium-coupled neutral amino acid transporter), member 10			-1.46
nve:NEMVE_v1g192163	K14709	SLC39 (zinc transporter), member 1/2/3	1.39		
bfo:BRAFLDRAFT_97943	K14718	SLC39 (zinc transporter), member 12;	1.71		
acs:100551962	K14720	SLC39 (zinc transporter), member 14	1.86		
aga:AgaP_AGAP011388	K14711	SLC39 (zinc transporter), member 5	-1.44		
isc:IscW_ISCW009278	K13862	SLC4 (sodium borate transporter), member 11	2.54		-1.37
spu:579418	K13862	SLC4 (sodium borate transporter), member 11	1.85		
nve:NEMVE_v1g106713	K13862	SLC4 (sodium borate transporter), member 11			-1.46
cin:100180760	K15377	SLC44 (choline transporter-like protein), member 2/4/5	1.26	-1.50	
tad:TRIADDRAFT_53446	K15377	SLC44 (choline transporter-like protein), member 2/4/5	1.90		
hmg:100211397	K14613	SLC46 (folate transporter), member 1/3	-8.81		1.68
nve:NEMVE_v1g163024	K14383	SLC5 (sodium/myo-inositol cotransporter), member 3	1.58		
bfo:BRAFLDRAFT_213839	K14388	SLC5 (sodium-coupled monocarboxylate transporter), member 8/12;	2.35		
oaa:100085055	K14386	SLC5 (sodium-dependent multivitamin transporter), member 6;	1.62		
rno:64522	K14382	SLC5 (sodium/glucose cotransporter), member 2		-1.07	
mmu:246787	K14382	SLC5 (sodium/glucose cotransporter), member 2		-1.04	
nve:NEMVE_v1g79785	K05048	SLC6 (neurotransmitter transporter, amino acid/orphan) member 15/16/17/18/20	1.80		

nve:NEMVE_v1g236066	K05034	SLC6 (neurotransmitter transporter, GABA) member 1;	1.23	-1.39	-2.86
cqu:CpipJ_CPIJ007997	K05037	SLC6 (neurotransmitter transporter, serotonin) member 4	1.90		-1.37
rno:64554	K13864	SLC7 (cationic amino acid transporter), member 2		-1.04	-1.34

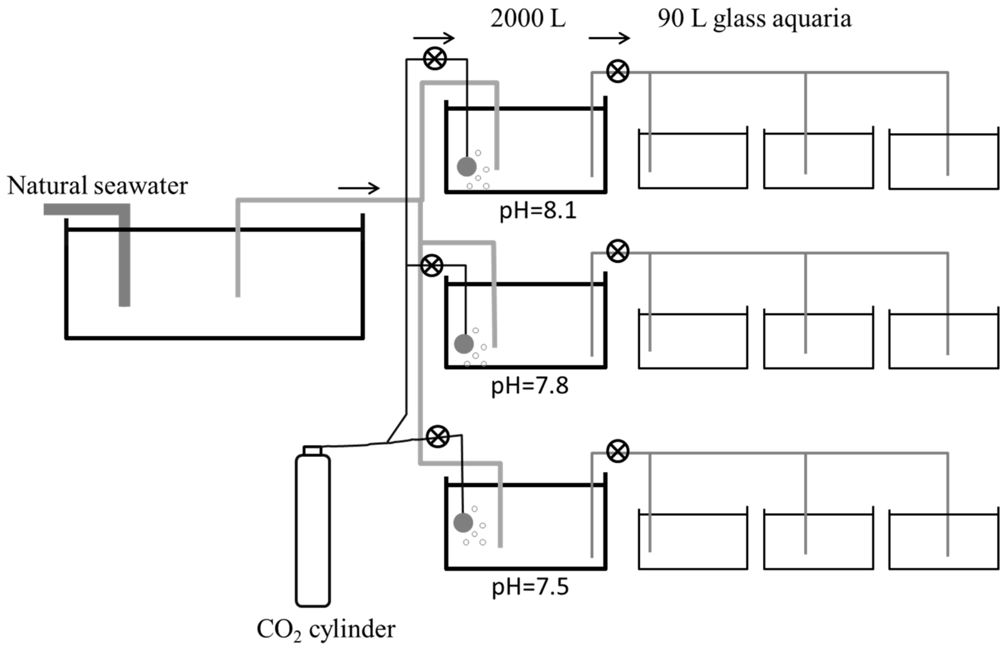

Fig. S1. Experimental design. Seawater from Sanya Bay was pumped through a pipeline into three 2000 L tanks. The natural seawater enriched with different pCO_2 levels was redistributed into nine 90 L glass aquaria from these three 2000 L tanks. Larvae were incubated in each 90 L glass aquarium in three treatments of different pCO_2 levels (390, 700, and 1200 μatm).
